# Trional

**Published:** 1894-04

**Authors:** 


### Trional.

Keppers, in a thesis read this summer at Wurzburg, draws the following conclusions from his experience of trional: It is a rapid hypnotic, especially useful in mental excitement. The headache which occasionally follows on its use quickly disappears. Thirty grains is a full dose. Disorders of the digestion seldom follow its use. Cardiac troubles contra-indicate its use. The anhidrotic action of the chemical is very marked; 3 to 4 grains arrest profuse perspiration. T. W. H.

*The Cincinnati Lancet-Clinic.*

